# Challenges experienced by patients with hypertension in Ghana: A qualitative inquiry

**DOI:** 10.1371/journal.pone.0250355

**Published:** 2021-05-06

**Authors:** Fidelis Atibila, Gill Ten Hoor, Emmanuel Timmy Donkoh, Gerjo Kok

**Affiliations:** 1 Valley View University, Techiman-Bono East Region, Ghana; 2 Department of Works and Social Psychology, Maastricht University, Maastricht, The Netherlands; 3 Department of Basic and Applied Biology, University of Energy and Natural Resources, UENR Sunyani, Sunyani, Ghana; 4 Professor Emeritus of Applied Psychology, Department of Works and Social Psychology Maastricht University, Maastricht, the Netherland; University of Sheffield, UNITED KINGDOM

## Abstract

**Background:**

Hypertension (HPT) is an essential public health problem affecting both lower and middle-income countries disproportionately. Evidence suggests that HPT is the leading risk factor for cardiovascular diseases and chronic kidney disease. Yet, challenges faced by patients with HPT in Ghana are not sufficiently explored. This study, documents the challenges patients with HPT face in Ghana.

**Methods:**

We used an explorative descriptive qualitative design. Face-to-face in-depth interviews were conducted with 15 patients with HPT. Interviews were recorded and transcribed verbatim. A thematic content analysis procedure was followed to analyse the data.

**Results:**

Four main themes emerged from interviews; three of which pertained to dimensions of challenges and a fourth which pertained to coping strategies. These include: [1] impairment in physical activities and mobility constraints [2]. Psychological challenges such as suicidal ideations, sadness, fear, anxiety, and reduced sexual affection [3]. Socio-economic challenges identified include loss of friends and social network, difficulty in job demands, and financial burden, and [4] coping strategies such as health system support, social support, and religiosity were identified.

**Conclusion:**

Patients with HPT experience an array of challenges. We suggest that health care facilities incorporate post HPT diagnosis counseling sessions for HPT patients in the study area. Also, the National Health Insurance Authority (NHIA) should re-examine their scope of services; thus, drugs, laboratory services, and electrocardiogram services to avoid the issue of co-payment. Collaboration between healthcare professionals and family relations of patients with HPT ought to also be strengthened to ensure optimal care.

## Background

Hypertension (HPT) is a significant public health challenge [1], affecting about 1.39 billion individuals (31% of all adults) worldwide [2]. HPT is defined as an elevated blood pressure greater than 140/90mmHg and an important treatable cause of cardiovascular (CVD) morbidity and mortality [3]. The World Health Organization (WHO) classifies HPT as a silent killer because it often does not show any warning signs and symptoms. Nevertheless, when signs and symptoms occurs, it manifests as headache, nosebleed, muscle weakness, dizziness, anxiety, palpitations and confusion. Estimates by the WHO [4] indicates that about 7.5 million deaths and 57 million disability-adjusted life years lost are attributable to HPT. However, Africa is disproportionally affected by the burden of HPT compared to other regions [4]. Specifically, HPT prevalence among men and women in Africa is over 40% compared to 35% of both sexes in the Americas [4]. In addition to the high burden of HPT, some patients with HPT are undiagnosed, untreated, or inadequately treated [5–7]. This is exemplified by a study conducted in three poor urban communities in the Greater Accra region of Ghana that showed that, in spite of HPT prevalence rate of 28.3%, only 7.4% of the participants included in the study were aware of their condition, 4% were on antihypertensive medicines, and 3.5% had their blood pressure (BP) under control [8]. Consequently, this phenomenon leads to chronic kidney disease (CKD), heart failure, ischaemic and haemorrhagic stroke and premature mortalities [3].

Extant literature has shown that HPT is a significant risk factor for cardiovascular diseases (CVD) (e.g., coronary heart diseases, ischemic heart diseases, and stroke), accounting for at least 45% of CVD related deaths [9, 10]. Other studies have also implicated genetic predisposition, race, age, psychological stress, cultural beliefs, socio-economic, and environmental factors as essential risk factors for HPT [2, 11]. Similarly, alcohol consumption, abdominal obesity, stress, and tobacco smoking have been identified as significant predictors for HPT in several countries [12, 13], including Ghana [14]. Unlike in the past when HPT was linked to the elderly and the rich, recent studies have demonstrated high prominence of HPT among children and young adolescents as well [15, 16].

Ferrell (1994) highlights a wide range of challenges that people with chronic conditions experience [17]. These include physical (i.e., functional activities, signs, and symptoms, psychological (anxiety, fear, and depression), social (appearance, financial burden, relationship, and affection/sexual function) and spiritual (hope, despair, religiosity, and inner strengths) aspects of the human beings [18, 19]. Similarly, diagnosis of HPT also presents some of these challenges to the patients [20]. For example, studies conducted in countries such as United States of America (USA), and Australia documented that patients with HPT suffer various forms of psychological, emotional, and social challenges [21, 22]. Notable among them are stress, depressive symptoms, and anxiety [22]. What appears to be missing in the literature is the challenges faced by patients with HPT following diagnosis. Understanding the challenges of these patients in Ghana where the prevalence of HPT is high (i.e., prevalence between 13.1% and 28%) [23–25] would assist in developing a comprehensive care to improve their wellbeing. Another important component of care for patients with HPT is how they cope with the disease condition. Explicitly, coping refers to a cognitive and behavioural effort made to reduce or tolerate an internal or external demand created by stressful situation [26]. The ability of an individual to cope is often dependent on the resources available, thus physical (health and energy) social (family, friends and social network), financial [26]. According to Lazarus and Folkman (1984) there are two main types of coping mechanisms: those aimed at resolving the stressful encounter (problem- focused) and those utilized to regulate unpleasant emotions that may arise during the encounter (emotion-focused) [27]. A prior study established a positive association between emotion-oriented coping strategies with reduced severity of HPT [28]. Considering that most studies in Ghana document HPT prevalence, risk factors, knowledge, and treatment [24, 29–31], this study explored the challenges patients with HPT face and how they cope with the disease condition in a selected municipality in the Bono region of Ghana.

## Methods

### Study design and setting

This study employed a qualitative explorative descriptive design [32]. The design was deemed appropriate considering that little is known about the challenges of HPT in the Ghanaian context. The study was conducted in Dormaa Presbyterian Hospital (HPT clinic). The hospital is part of the Christian Health Association network located in the Dormaa municipality in the western part of the Bono Region. It is bound in the north by the Jaman South district and in the east by the Dormaa East district, in the south and south-east by Asunafo and Asutifi districts respectively, in the west and south-west by Dormaa West and in the west and north-west by La Cote d’Ivoire. The municipal capital is Dormaa Ahenkro, located about 80 kilometers west of the regional capital, Sunyani. The municipality has a total land area of 1,210.28 square kilometers, which is about three percent of the total land area of Bono Region [33]. It is a district Hospital with a bed capacity of 188. The HPT clinic is run three (3) days in a week (Monday, Wednesday, and Thursday) which attends to about ninety (90) HPT patients on clinic days. The services offered at the HPT clinic include specialist consultation, electrocardiogram (ECG) services, and health education.

### Participants and recruitment

The participants comprised of 15 patients diagnosed with HPT. We included patients who were 18 years and above who attend the HPT clinic. Patients with HPT who had co-morbidities, such as Diabetes Mellitus, were not included. A purposive sampling technique was employed. This technique allowed for the selection of participants with the appropriate experiences in HPT to give the desired information about the phenomenon under study [34, 35]. The purpose of the study was discussed with the staff. Two of the nurses working in the unit assisted in the recruitment process. The nurses reviewed the patient’s folders, selected the potential participants, and introduced them to the research team. Potential participants were briefed on the purpose of the study, the voluntary nature, and confidentiality arrangements. Out of twenty (20) participants approached, five (5) did not agree to participate: three (3) of the participants cited time constraints, and the other two did not provide any reason. Those who did not participate in the study did not have their health care affected. Data saturation occurred after the 13th interview. However, two (2) additional interviews were conducted to determine whether any new issue may emerge. Consistent with the previous themes and subthemes, no new information emerged.

### Data collection procedure

Data collection took place between December 2019 and February 2020. Participants were contacted two days before the interview via telephone to remind them and to confirm the venue and time. On the scheduled days, FA (Master of Public Health in Health Promotion/ PhD candidate) a male registered nurse with principal nursing officer grade and experience in qualitative research conducted in-depth one-to-one interviews. Informed consent (including permission to audio record the interview session) was thumb-printed or signed by the participants. The PhD candidate started the interview by introducing general conversation to create rapport before explaining the purpose of the study. Participants were also assured of anonymity and confidentiality of information given in the study. This helped the participants to relax before interviews were conducted using a semi-structured interview guide. In addition, demographic characteristics were taken, including age, gender, occupation, and religion. A total of 15 participants took part in the study. Eight interviews were conducted in private at the nurses’ station, and the remaining seven had their interviews done at their private residence, mostly under trees. Five of the interviews were conducted in ‘Twi’ (local dialect), and ten (10) were done in English. Field notes were taken during the interview process through keen observation of the non-verbal cues expressed by respondents. Some of these non verbal cues were a state of silence by some of the participants when they were recounting their experiences. The interview sessions lasted between 45–60 minutes each and were audio-recorded. Data saturation was reached after the interview of the 13th participant since no new and unique themes emerged during further interviews [36, 37].

### Research instrument

A semi-structured interview guide was developed based on empirical literature on hypertension [2, 38] and with the guidance of an expert in the area of qualitative research who is also the second author. Subsequently, two people with hypertension were recruited from the District Hospital in Wamfie, a neighbouring town to pilot the interview guide. The pilot study helped in strengthening the guide to elicit appropriate information to answer the questions. The topics explored during the interview were (1) whether people with HPT experience some physical challenges (2) psychological challenges (3) socio-economic challenges (4), and coping strategies. The interview guide can be found in S1 File.

### Data management, analysis and reporting

The data were processed using Nvivo software version 10.0 [39]. Thematic content analysis procedure was followed for the analyses of the data. The first author familiarized himself with the data by listening to the audio and subsequently transcribed the data. Following the transcription, two experts who were fluent in both the Twi and English languages translated the transcribed anonymized Twi interviews to English using the principle of “back to back” translation. This was done to ensure that the content meaning of the data were maintained. Third, the transcripts were systematically coded by the first and the third author, after which, the codes were collated into potential themes. These themes were discussed by the authors in a series of meetings until consensus was reached on the final themes [40]. The consolidated criteria for reporting qualitative research (COREQ) was followed for this report [41].

### Rigor

A qualitative finding is considered trustworthy if it is credible, transferable, dependable, and confirmable [42, 43]. Credibility is used to assess the level to which the data reflects the reality and is representative of the participants’ views [44] At the end of each interview the PhD candidate cross-checked to appreciate fully and adequately present the participants’ stories to ensure credibility. Dependability refers to an assessment of the quality of the integrated processes of data collection, data analysis, and theory generation that can be audited [45]. This was achieved by the provision of a detailed description of the research methodology (recruitment process, data collection, data analysis). Transferability refers to how the study results could be used in other situations and contexts [46]. To ensure this, a detailed and comprehensive description of the study setting was provided. Confirmability was ensured by keeping the field notes and voice records.

### Ethics

Ethical clearance was obtained from the Institutional Review Board of the Christian Health Association of Ghana (approval number PIN -15052019). Also, permission was granted by the management of Dormaa Presbyterian Hospital and written informed voluntary consent was provided by the participants. After being briefed on study procedures, participants indicated their willingness for inclusion by filling out a consent form and appending their signature or thumb-printing same. All personal records of participants were anonymized and participants were free to decline to participate in the study without consequence to healthcare.

## Results

### Demographic characteristics

The 15 study participants were made up of 8 males and 7 females, aged between 36 years and 69 years. The number of years of marriage by participants was from 5 years to 31 years. The number of years participants have lived with HPT was from two (2) years to (10) years. In terms of levels of education of participants, 2 had no formal education, and the remaining 13 had attained various levels of education. Amongst the 15 participants, one was a widow, and the remaining fourteen were married. Eleven (11) of the participants were Christians, and four (4) were Muslims. In terms of employment status, four of the participants were teachers, three were traders, three were farmers, and two were cleaners. Two of the participants were not employed. Participants ‘current treatments ranged from antihypertensive and dietary management or both. Detailed characteristics of participants are shown in S1 Table.

### Main findings

Four main themes and twelve sub-themes were generated from the data. These themes and their sub-themes are presented in the Table 1.

**Table 1:** Summary of themes and sub-themes from the transcribed data.

Themes	Sub-themes
1. Impairment in physical activities	• Limited participation in household routines
	• Mobility constraints
2. Psychological challenges	• Suicidal ideations
	• Sadness
	• Fear and anxiety
	• Reduced sexual affection
3. Socio-economic factors	• Loss of friends and social network
	• Difficulties with job demand
	• Financial burden
4. Coping strategies	• Health system support
	• Social support
	• Religiosity

### Impairment in physical activities

Participants expressed their inability to perform some activities of daily living. The sub-themes identified were 1) limited participation in household routines and 2) mobility constraints.

### Limited participation in household routines

A number of the participants expressed their inability to wash their unclean clothes due to excessive weakness and dizziness associated with HPT. As a result, the participants decided not to wear many clothes to decrease the number of clothes that required laundry. This was because participants depended on their relations or sought outside support for laundry. This is exemplified by narratives by two participants:

*“Hmmm!* *(sighing) Even washing my dirty clothes is a challenge*. *I sometimes have to call some of my neighbours to help me*. *Because of my condition*, *I don’t wear so many clothes*, *so it takes about two weeks or a month before I find somebody to come and help me wash*.*” (F012-Age-58-Number of years of diagnosis (NYD)-7)*

*“When my husband comes to visit*, *he always washes my clothes*, *can you imagine that*? *Hypertension is a bad disease*. *I always feel so weak that I am unable to do most of the basic things I used to do without stress*.*” (M002-Age-45-NYD-5)*

Others also said they relied on family relatives, including sisters-in-law, to wash their dirty clothes. Some participants said:

*“These days*, *anytime my clothes are dirty*, *my husband’s sister washes for me*. *She always asks for my dirty clothes when she is doing the laundry*. *What I do is that I don’t wear so many clothes*. *I wear only this dress all the time; if I wear so many clothes*, *it will be a burden on her*.*” (F004-Age-50-NYD-4)*

*“As for my laundry*, *my younger sister does the washing for me*. *Though she is a family member*, *I become embarrassed because it is not suitable for someone to wash your under-garments*. *People around me find it difficult to understand my behavior now*.*” (F010-Age-41-NYD-3)*

In addition, some of the participants lamented that they were unable to bath and groom themselves as they used to do because they always feel weak and needed support. Participants stated that they were assisted by their wives, husbands, and daughters to bath and groom.

*“I cannot do anything now because my heart is always beating very fast when I engage in any strenuous activities*. *My heart was not like this initially*. *There are days that I can’t bath on my own without someone assisting me in doing so*.*” (M013-Age-60-NYD-4)*

Conversely, a participant cited that he had nobody to assist in bathing and grooming and therefore tried to bath and groom himself even when he is very weak. He lamented:

*“I live alone with no children around me*. *Sometimes the body weakness and dizziness are so severe that I have to skip a proper bath for three days and only wash my face and my armpit just to smell nice*. *Even with that*, *if I keep long in the bathroom*, *I feel like falling*. *I have no one to support me because my wife is out of town*. *I have to always manage like that*, *and it is very difficult for those of us who have nobody around to care for us*.*” (M007-Age-53-NYD-3)*

Also, some participants stated that they could not cook due to persistent weakness and severe headaches. Participants cited that they sometimes sleep in hunger.

*“I was not able to cook last night*. *Because I felt very weak and was also experiencing severe headaches*. *Deep in the night*, *I felt so hungry that I had to take some banana and drank some water before I could sleep*. *The following night I felt the same way and had to take banana again*.*” (F006-Age-55-NYD-4)*

*“Sometimes*, *when I cast my mind back to the things I used to do especially cooking for my family*, *I become sad and cry*. *I cannot imagine myself depending on my neighbours to buy my foodstuff and other things that I feel like buying*. *I feel helpless*, *and sometimes I stay hungry when no one is around to be sent or to cook for me*.*” (F011-Age-56-NYD-5)*

### Mobility constraints

Some participants reported mobility constraints due to persistent weakness, palpitations, headaches, and reduced strength because of the side effect of the drugs. As a result of that, they cannot go about some routine activities, thus, going to the market, grocery shops, work, and church. A number of the participants expressed this as worrying, particularly having to depend on others for their livelihood.

*“I am always feeling so weak and easily get tired when I walk for a short distance*. *As a result of that I am unable to go to the market*, *so I always make people around to buy the food items in bulk*.*” (F010-Age-41-NYD3)*

*“I cannot go anywhere*, *to the farm*, *church*, *or anyplace I used to go*. *Walking is very difficult for me now*. *I feel tiredness and weakness in my left leg and my left hand*. *Sometimes I have to be supported when I am getting up*, *even when getting into a chartered taxi to the hospital; people have to support me*. *I am now confined to only my home because of this disease*.*” (M003-Age- 61-NYD-5)*

### Psychological challenges

Psychological challenges refer to mental and emotional difficulties HPT patients face. Four sub-themes emerged from the data: suicidal ideation, sadness, fear and anxiety, and reduced sexual affection.

### Suicidal ideation

Some participants cited that they contemplate suicide because they believe HPT cannot be cured. Participants expressed the desire to end their lives rather than going through the anguish and the suffering associated with an incurable illness (HPT). Some of the participants felt that taking the medications throughout their life was going to be too burdensome. Others thought they were too young to develop HPT.

“*I cannot sleep in the night*, *and I don’t even know what to do*. *This world is a bitter place with incurable diseases*. *When I think of the fact that I developed this disease at this young age*, *and no drug can cure it*, *I sometimes feel like killing myself in time past*. *Hmmm (heaves a sigh) it has not been easy for me at all*.*” (M001-Age-36-NYD-2)*

*“I am always so weak*, *constant headache*, *and very fast heartbeat with difficulty to sleep at night*, *so sometimes I think killing myself will end all the suffering*. *It is better to die than to live with an illness which is not curable*. *I have to take drugs like that until I die*.*” (M005-Age-38-NYD-3)*

Others felt that their over reliance on family members for support to engage in their daily routines makes them feel that suicide is a better option. According to them, the situation is much compounded when they have to attend to a physician appointment in the hospital.

*“As for me*, *I prefer to die and end it than to suffer like this*. *Anytime I am coming for review*, *they have to support me into the car because the stroke has affected my walking*. *I feel I am so much a burden to them (relatives)*. *My son*, *it is not easy*.*” (M014-Age-69-NYD-10)*

### Sadness

Some of the participants expressed sadness because they felt they were too young to develop HPT. The participants who were younger perceived that HPT is a disease for the elderly and therefore considered their disease condition as an unfortunate event for their age.

*“My brother*, *I have been thinking about why I should develop hypertension at this younger age*. *This means that I have to be on medication for the rest of my life*, *and this always makes me so sad*.*” (M001-Age-36-NYD-2)*

Others also attributed the experience of sadness to their inability to afford the high cost of HPT medications that were not covered by the National Health Insurance Scheme.

*“It’s normal to be sad when you have been given drugs to buy but no money to take care of the cost*. *It leaves you very sad*.*” (F008-Age-38-NYD-2)*

Some were also concerned about the future of their children due to the unknown outcome of HPT. Some of the participants said they often feel sad because of fear of possible complications of HPT that could stress up their children.

*“When I see my children coming*, *I pretend and smile like everything is ok; but inside me*, *I am a different person*, *always thinking of the future of my children*, *because if I get any complication like stroke now*, *how do I take care of them*, *and this makes me so sad*.*” (M007-Age-53-NYD-3)*

*“A lot of things make me sad*. *Sometimes if I see a healthier younger woman of my age going about her normal activities*, *I frequently ask*, *why me*? *Why can’t I go up and down like them*? *Where did I go wrong to deserve this to punish my children*?*” (F008-Age-38-NYD-2)*

### Fear and anxiety

An account by some participants indicated that they feared they may die from HPT complications and were concerned about where they will go after death. Hearing about the death of friends and also remembering the death of a family member from complications of HPT such as stroke and kidney diseases, caused anxiety.

*“Hmmm*, *anytime I think of my death*, *I become afraid and anxious*. *I can’t imagine dying at this age and leaving my family behind*. *This puts so much fear in me because the children are quite young and who will take care of them when I am not there*.*” (F009-Age-61-NYD-6)*

*“I have the fear that I may die*. *When I was diagnosed with hypertension*, *I cried because my aunty died from the complication of this same condition (HPT)*. *Sometimes I am anxious about what will happen to me some days to come*. *I saw my aunty suffer a stroke as a result of HPT and eventually died” (F006-Age-NYD-4)*.

According to the participants, the information on HPT shared on the media that suggests that HPT is incurable persistently reminded them of their likelihood of dying, thereby intensifying their anxiety and fear.

*“I am always anxious and afraid because of the media report I hear about hypertension*. *They always say once you are diagnosed with HPT; you cannot be treated in the hospital*. *And you will eventually get a stroke and die*. *Anytime I hear this kind of information from the radio stations I get frightened*.*” (M015-Age-58-NYD-3)*.

#### Reduced sexual affection

Most participants said that they experience reduced sexual affection following the intake of the prescribed anti-hypertensive medications. The reduced sexual affection was said to have marred the relationship with their partners. Thus, consequently affecting their marriages.

*“I don’t have any urge for sex since I started taking the medication*. *I don’t have feelings for my wife*, *and I cannot remember the last time I made love with my wife*.*” (M007-Age-53-NYD-3)*

The majority of the male participants asserted that their reduced urge for sex is disturbing them because their partners seem not to understand the problem. That most of their partners feel that they are having extra-marital affairs. The numerous complaints were said to be a bother by all the male participants who were married.

*“Things have changed*, *I used to disturb my wife for sex all the time*, *but now the urge is no more because of the constant palpitations I have*, *I don’t understand what is happening*, *my wife is always complaining*!*” (M002-Age-45-NYD-5)*

*“(Laughs)…I have forgotten about that one*. *For sexual feelings*, *I have made up my mind because ever since I started taking the drugs*, *I don’t have the desire for sex*. *She has been complaining and even suggested that I go to see a doctor for treatment*.*” (M005-Age-38-NYD-3)*

### Socio-economic factors

Participants reported their inability to attend social functions, loss of friends, financial burden, challenges with job demands, and special diet as some of the difficulties they experience following their diagnosis with HPT.

### Loss of friends and social network

Participants narrated that they have lost most of their friends. This is because they are not able to attend important social gatherings such as funeral events, weddings, and naming ceremonies as they used to do. The commonest reason given by almost all the participants was tiredness.

*“I am always at home because when I walk small*, *I get tired*. *These days I usually don’t attend most of the activities that go on in this area*, *and because of that*, *I have lost most of my friends*. *I don’t get to see them*, *and they don’t come to visit me either*.*” (F008-Age-38-NYD-2)*

Some of the participants indicated that they felt their existing relationships were strained by their HPT status: especially when they were unable to perform social and marital roles as they previously did. Inability to return visits was one cause of strain on friendships.

*“Now I don’t go to our usual joints to meet my friends*: *for almost one year now*, *because I easily get tired when I walk*, *I mostly stay at home*. *But my friends don’t visit me at home either*. *Some of them even think I am just lazy*.*” (M002-Age-45-NYD-5)*

*“Master*, *now my friends don’t come to me because I have not been visiting them these days because I don’t feel well*. *Not even one person since I felt sick*. *They have all abandoned me*.*” (M001-Age-36-NYD-2)*

### Financial burden

Some of the participants reported a financial burden. Participants complained of financial constraints as a result of the costs they incur when they visit the hospital. These include costs of transportation, laboratory investigations, and the cost of medications. Most participants mentioned that they had to pay top-up (co-payment) before they are served the prescribed drugs though they were subscribers of the National Health Insurance Scheme (NHIS).

“At times, I don’t get the drugs in the hospital, and it is also expensive to buy the drugs outside the hospital. There are times that the drug is available in the hospital, but you have to pay an extra amount called top-up when you are an NHIS subscriber before you are given the medication. This brings about a substantial financial burden on me.” (F011-Age-NYD-5)

*“Hmmm*, *as for my finances*, *it has affected me in so many ways*. *Anytime I go to collect drugs from the hospital*, *they always demand a top-up before I am served the medications though I am an NHIS subscriber*. *Everything in Ghana now is all about money*.*” (M014-Age-69-NYD-10)*

Others mentioned that to meet their monthly drug supply, they have to resort to friends for financial support.

*“I sometimes have to buy some of my prescribed medicines from the drug store (pharmacy) because it is not available in the hospital*, *and when I don’t have enough money*, *I have to borrow from someone and pay later*.*” (F012-Age-58-NYD-7)*

Also, one of the participants said that he has run into debt because of the cost of drugs and laboratory investigations.

*“As for the cost of treatment*, *it’s not easy at all*. *I have run into debt*. *Because when it was severe*, *we had to go to a regional hospital on referral to continue treatment*. *My money got finished at a point*, *and I had to borrow money to do all the laboratory investigations requested*. *They said the insurance does not cover some of the laboratory investigations*, *so I had to pay*.*” (F009-Age-61-NYD-6)*

In addition, a participant narrated that he is going through financial difficulties because they have to always prepare a separate meal for him, and this comes with additional cost.

*“It is always difficult for me when it comes to my treatment because the doctors advised that I reduce my salt intake*. *So these days*, *my family has to prepare a different meal for me*, *and this also comes with extra costs*.*” (F006-Age-55-NYD-4)*

However, some participants did not bear the cost of treatment. Either the family members supported settling their hospital bills, or these were paid by private insurance companies.

*“In terms of money*, *since I started going to the hospital*, *I haven’t had any problem*. *Things are going on well with me because the family has been very supportive financially*.*” (M002-Age-45-NYD-5)*

*“All this while the private insurance company always pays the cost of treatment*, *and because of that*, *things are moving well with me*. *I don’t pay bills at the hospital when I go for treatment and reviews*. *The bills are always submitted to private insurance*, *and they pay on my behalf*.*” (M015-Age-58-NYD-3)*

### Challenges with job demands

Some participants said they had to change their work schedules, and others indicated that they absented themselves from work frequently because of the disease condition. This was said to have created friction between the participants and their supervisors.

*“These days I don’t regularly go to work because I have to always go for checkups on some of the days*. *This is affecting my productivity at the workplace*. *Because of that*, *my boss is always complaining and even threatening to lay me off*.*” (F006-Age-55-NYD-4)*

For those operating their businesses, they lost some of their customers. This impacted negatively on their relationship with their supervisors and customers hence a decrease in their financial inflows.

*“The problem it has brought is that it has stopped me from doing my usual rounds*. *Typically*, *someone can call me that he or she needs an item to buy*. *I often deliver the items quickly*. *But these days*, *there are times that I have to reschedule the person to meet me at another time because I may be at the hospital because of my regular check-up*, *and this makes me lose my regular customers*.*” (M007-Age-53-NYD-3)*

### Coping strategies

Under coping strategies, three sub-themes were identified: 1) health system support, 2) social support, and 3) religiosity.

### Health system support

Almost all the participants indicated that the nurses, doctors, dietitians, and other health care professionals were very supportive. Participants, therefore, commended nurses and doctors for the teachings and assistance they offered them when they were emotionally disturbed. Some of the participants indicated that the positive attitude and support of health care professionals aided them in coping with the HPT diagnosis.

*“The health professionals have been very supportive in my treatment*. *They relate to me nicely*, *and some of them often give us health education on HPT*, *and I think they need to be applauded*. *Some even give me words of encouragement when they see that I am not looking cheerful*, *and this helps me to cope with the disease*.*” (M014-Age-69-NYD-10)*

*“As for me*, *I will be telling a lie if I say I have any problem with the staff of the hospital*. *They take time to teach us on some of the things we need to do to stay healthy and the need for regular reviews*.*” (M015-Age-58-NYD-3)*

Despite the positive attitude of some of the health care professionals reported above, some participants expressed negative experiences.

*“My major problem is with the doctors of the hospital*. *They don’t spend time listening to our complaints*. *The moment you start to tell them about your problem*, *they will tell you that they are done with you*, *so go for drugs*.*” (F009-Age-61-NYD-6)*

Others also mentioned that the time spend for retrieval of their health record book is too long.

*“The problem I have is concerning the records office*, *you will sit down for long*, *and nobody will tell you anything*, *they shout at us as if we are children*. *The last time I nearly went back home because I sat for long and I was not getting my card number until one man came and told us the internet was down*.*” (M013-Age-60-NYD-4)*

### Social support

Most of the participants stated that they received physical, emotional, social, and financial support from their spouses:

*“My wife is a perfect woman; she is always supporting and encouraging me all the time*, *and this makes me have a positive attitude*. *I think if you develop this condition and your wife doesn’t support you emotionally*, *you will die early*.*” (M005-Age-38-NYD-3)*

*“I am very grateful to God for my husband*. *He has helped me so much*. *He makes sure that he provides for me financially and even takes care of the home keeping*. *Our children are grown up*, *so we live alone*. *Truly*, *I wouldn’t have survived without him*. *We pray together*, *and he always encourages me to have faith in God*.*” (F004-Age-50-NYD-4)*

Some participants also reported that family members were very instrumental in terms of providing emotional and financial support, and that aided patients with HPT to cope. Participants narrated as follows:

*“My sister has been really supportive financially when it comes to payment of my hospital bills*. *She is always with me encouraging me and that gives me a lot of strength to cope with this challenging disease*. *At times she comes around to do cleaning and even cooks for me*.*”* (M001-Age-36-NYD-2)

*“My family and friends have been very supportive ever since I was diagnosed with the disease (HPT)*. *The extended family members visit me regularly*, *and some of them also support me financially*.*” (F012-Age-58-NYD-7)*

On the contrary, participants who had a history of marital disputes before the diagnosis of HPT did not receive emotional support from their partners:

*“My husband only buys food for me*, *but he does not have time for me*. *No affection*, *no attention*, *and care*. *He is always busy all the time and comes home very late” (F006-Age-55-NYD-4)*

*“When I am not feeling well*, *as a husband*, *I expect him to spend meaningful time with me*. *At least*, *be close to me; hold me and talk with me and encourage me with words from the Quran*. *But he is always going to his second wife and leaves me to suffer alone*.*” (F011-Age-56-NYD-5)*.

#### Religiosity

Religiosity is an essential part of the Ghanaian culture, which influences beliefs and attitudes towards illness and difficult situations. In the context of this study, religiosity was characterized by participants’ faith in God and the adherence to practices such as prayer and fasting. Participants described religiosity as one of the critical resources, which helped them to deal with the condition.

*“Prayer is the key*, *and it has helped me*. *Sometimes it surprises me how God uses my prayers to make me move on*. *If not for constant prayers*, *I would have been dead by now*. *This sickness can easily kill you*, *but God has been good to me so far*.*” (M015-Age-58-NYD-3)*

*“It has made my faith stronger*. *I am much closer to God*, *and I believe this will bring an improvement in my disease situation*, *so I need to go on believing in God*. *I have the faith that I will get better one day*, *and this encourages me to move on*.*” (M013-Age-60-NYD-4)*

Others indicated that their religious leaders visit them in their home to offer spiritual support when they are unable to attend physical church services.

*“I have turned my house to be my chapel because I cannot go to church as often as I used to*. *And when you have a disease that has no cure*, *you have to turn to God for healing*. *My pastor and church leaders also visit me in my house regularly to pray with me; this helps some of us to cope with the disease*.*” (F008-Age-38-NYD-2)*

## Discussion

This study explored the challenges patients with hypertension face in Ghana. Broadly, challenges affecting patients with hypertension can be resolved into three categories; 1) impairment in physical activities, 2) psychological problems and 3) socioeconomic factors. The coping strategies used by the participants were also explored. Although these identified challenges were similar to challenges as found by others, the implications within the Ghanaian culture are different.

Related to the impairment in physical activities, our participants highlighted the difficulties they encounter when performing routine activities such as ensuring personal hygiene, going to market, grocery shops, and cooking. This is in line with findings from [2, 47, 48]. However, some of the female participants felt unhappy that they had to depend on others to do their laundry and cooking, a role that is entrenched as a feminine duty per the Ghanaian cultural system. It can also be contended that dependency on the part of HPT patients could present some form of psychological effects on the participants such as depression.

Consistent with Cuevas et al., [22], our participants indicated psychological problems such as sadness, suicidal tendencies, fear, and anxiety, probably resulting from misconceptions about HPT and its complications [49]. These misconceptions, largely broadcast on television and radio stations by untrained health professionals seeking to portray HPT as an unmanageable condition persistently reminded them of their likelihood of dying from the complications of HPT, thereby increasing their level of anxiety and fear. This phenomenon is quite common in the Ghanaian context. This implies that health care professionals managing people with HPT should provide proper health education and post-diagnosis counseling to allay fears and anxieties regarding the disease process to help avert these misconceptions propagated by untrained health professional.

Also reduced sexual affection was highlighted as a problem for patients with HPT, and a serious challenge in their relationships. This experience could be the result of the side effects of HPT medications and the disease process [20, 50, 51]. In the Ghanaian context, sex has a profound impact in a marital relationship, and inability to satisfy your partner is often perceived to mean cheating or infidelity; hence can lead to marital breakdown.

Patients with HPT in Ghana also experience substantial financial burdens. There are high cost of medications and laboratory investigations, even though the majority were subscribers of the NHIA, there was a constant demand for top-up (co-payment). This could have serious implications for adherence to treatment and management of HPT and it contributes substantially to poor control and management of HPT [24, 52]. Most anti-hypertension drugs are readily available in the Ghanaian market, but not all medicines and laboratory investigations are catered for under the NHIA service scheme. Secondly, patients with HPT were advised to modify their diet to help control blood pressure. As a result, their families have to prepare two separate meals. This has additional financial implications to these families [53]. Thirdly, one unique finding was that some participants complained of having difficulties with their employers and customers because of their inability to go to work. Others cited that their frequent absence from work because of regular check-ups at the hospital has brought about dissatisfaction and low productivity at the workplace [54]. This could lead to possible job losses and hence a reduction in income of patients with HPT.

For coping and support, participants commended nurses and doctors for the teaching and assistance they offered them when visiting the facility. Some even indicated that the emotional support provided by health care professionals aided them in coping with HPT. Unexpectedly some patients expressed their misgivings about the short time doctors and other health professionals spend with them during consultation after long hours of waiting [55]. Support from health professionals should therefore be seen as therapeutic, and health care workers should try as much as possible to spend considerable time with patients during consultation to provide them much time for them to ask relevant questions regarding treatment regimen. This will also afford the health professional ample time to give the patients the needed information on the therapeutic regimen and review schedules.

Majority of participants reported receiving physical, emotional, and financial support from their partners and family members. Family support is indispensable and patients felt encouraged psychologically by spouses and family members helping them to cope with disease conditions [38, 56–58]. This new argument shows that HPT introduces an additional layer of stress to existing relationships which must be managed and suggests that patients with poorer family/marital relationships may be at increased risk of poorer psychological outcomes and may require additional support. Taking up roles and responsibilities by partners and family members in the Ghanaian setting is a value that is deeply ingrained within our society and helps coping with chronic conditions.

Lastly, most participants expressed that religiosity is a valuable resource that aided them in coping with the condition. Some reported that prayer, faith, and trust in God and visits by religious leaders contributed to their coping. Several authors have noted similar outcomes under similar circumstances where regardless of their religious beliefs, participants took solace in God [49, 59–61].

### Strengths and limitations of the study

The findings of this study should be viewed in light of some strengths and limitations. In terms of strength, this study is one of the few studies that report on the challenges experienced by patients with hypertension in Ghana and Sub-Saharan Africa. The explorative nature of the study assisted in the understanding of the phenomenon more broadly and in context. However, the findings may not be applicable to hypertensive patients with comorbidities such as diabetes mellitus and severe complications for which care protocols are different. The sample size of 15 participants limits the possibility of generalizability. An exploration of the spatio-temporal relationship between diagnosis of hypertension and mental health would be a useful follow-up. Also, a quantitative study would be beneficial in clarifying whether community management strategies emphasizing collaboration between caregivers and family members in the management of patients can improve care outcomes. Second, the collection of data on one occasion is limited to some extent because one cannot establish how long the themes would last. Third, recall bias may be an important limitation of this study considering that the participant had to recount their past experiences with the condition. Nevertheless, the findings can help health workers to design and implement interventions that address the mental and sexual health needs of patients with hypertension.

## Conclusion

This study highlights the challenges patients with HPT encounter in Ghana. Challenges such as suicidal ideation, cost of services/co-payment and reduced sexual affection were reported. It is recommended that post-diagnosis counselling for patients with HPT should be included in the formal care. The NHIA should also re-examine their scope of services for patients with HPT as well as the co-payment of some essential hypertensive medications, laboratory investigations, and radiology assessment in order to remove cost as a barrier to health seeking. The collaboration between healthcare professionals and family relations of patients with HPT ought to be strengthened to ensure optimal care.

## Supporting information

S1 File Interview guide. (DOCX)

S2 File Supporting data. (DOCX)

S1 Table General participants profile. (DOCX)

## Abbreviations

BP: Blood pressure
CVD: Cardiovascular diseases
DPH: Dormaa Presbyterian Hospital
ECG: Electrocardiogram
HPT: Hypertension
IRB-CHAG: Institutional Review Board-Christian Health Association of Ghana
NHIA: National Health Insurance Authority
NHIS: National Health Insurance Scheme
NYD: Number of years of diagnosis
JSS: Junior Secondary School
WHO: World Health Organisation
