# Supplementary material for: Challenges experienced by patients with hypertension in Ghana: A qualitative inquiry
Source: PLoS One. 2021 May 6;16(5):e0250355. doi: 10.1371/journal.pone.0250355 (PMC8101754; doi:10.1371/journal.pone.0250355)
Supplement: S1 File — (DOCX) [file pone.0250355.s001.docx]

**S1 File**

**Interview Guide**

**A) Background information of respondents**

1. Age ………………………
2. Gender……………………………………………….
3. Educational background…………………………….
4. Marital status…………………………………………
5. Religion……………………………………………….
6. Occupation……………………………………………
7. Number of years you have been living with hypertension………………………………

**B) Challenges of respondents**

1. Can you describe what happened before you visited the hospital?
2. What was your initial reaction when you were informed of being hypertensive?
3. Can you share with me some of your physical challenges after being diagnosed with HPT?

Probe

1. Can you tell me what happened after you were put on medications? Probe:

Side effects of drugs

5. How does this condition make you feel psychologically? Probe:

Sadness

Anxiety

Suicidal ideation

Fear of complications;

Unpleasant statement from friends and family, if any

6. How has this affected your social life? Probe

- Work

- Taking care of the children

- Family support

- Marriage

- Financial issues

-Church

7. What are some of the challenges of living with hypertension? Probe:

Financial issues e.g., Cost of medications

Beliefs systems e.g. church and other outdoor activities

Attitudes

Health system related (Health workers attitude, delays in seeking care)

8. Can you tell me how you are able to cope with your diagnosis and its accompanying challenges? Probe

9. How would you assess your encounter with the health care providers? Probe:

Interpersonal relationship

Communication

Attitudes

Health system-related (Health workers attitude, delays in seeking care)

10. Do you have anything to tell me aside what has been discussed?

**Thank you**
