# Supplementary material for: Challenges experienced by patients with hypertension in Ghana: A qualitative inquiry
Source: PLoS One. 2021 May 6;16(5):e0250355. doi: 10.1371/journal.pone.0250355 (PMC8101754; doi:10.1371/journal.pone.0250355)
Supplement: S2 File — (DOCX) [file pone.0250355.s002.docx]

| **THEMES** | **SUB-THEMES** | **ROOT CODES** | **QUOTES** |
| --- | --- | --- | --- |
| **Impairment in physical activities and mobility constraints** | •Limited participation in household routines  •Mobility constraints | - Incapacitation; domestic chores   Restricted lifestyle choices  Dependence; family support  Special social needs  Incapacitation; domestic chores  Dependence; social support  Embarrassment  Incapacitation; basic activities   - Weakness - Malaise - Helplessness - Hunger pangs - pain/immobilization - physical support - confinement - Insomnia | P012“Hmmm! Even washing my dirty clothes is a challenge. I sometimes have to call some of my neighbours to help me. Because of my condition, I don’t wear so many clothes, so it takes about two weeks or a month before I find somebody to come and help me wash.”    P002“When my husband comes to visit, he always washes my panties, can you imagine that? Hypertension is a bad disease. I always feel so weak that I am unable to do most of the basic things I used to do without stress.”  P004“These days, anytime my clothes are dirty, my husband’s sister washes for me. She always asks for my dirty clothes when she is doing the laundry. What I do is that I don’t wear so many clothes. I wear only this dress all the time; if I wear so many clothes, it will be a burden on her.”  P010“As for my laundry, my younger sister does the washing for me. Though she is a family member, I become embarrassed because it is not suitable for someone to wash your panties. People around me find it difficult to understand my behavior now.”  P013“I cannot do anything now because my heart is always beating very fast when I engage in any strenuous activities. My heart was not like this initially. There are days that I can’t bath on my own without someone assisting me in doing so.”  P006“I was not able to cook last night. Because I felt very weak and was also experiencing severe headaches. Deep in the night, I felt so hungry that I had to take some banana and drank some water before I could sleep. The following night I felt the same way and had to take banana again.”  P011“Sometimes, when I cast my mind back to the things I used to do especially cooking for my family, I become sad and cry. I cannot imagine myself depending on my neighbours to buy my foodstuff and other things that I feel like buying. I feel helpless, and sometimes I stay hungry when no one is around to be sent or to cook for me.”  P015“My brother, this disease is very challenging. As I talk to you now, I feel weakness all over because of that I am always indoors lying down. I cannot go to work nor do anything. How can a man like me be at home most of the time? Even Sundays I am unable to attend church service.”  P010“I am always feeling so weak and easily get tired when I walk for a short distance. As a result of that being unable to go to the market, so I always make people around to buy the food items in bulk.”  P003“I cannot go anywhere, to the farm, church, or anyplace I used to go. Walking is very difficult for me now. I feel tired and weakness in my left leg and my left hand. Sometimes I have to be supported when I am getting up, even when getting into a chartered taxi to the hospital; people have to support me in the taxi. I am now confined to only my home because of this disease.” |
| **Psychological challenges** | - Suicidal ideations - Sadness - Fear and anxiety - Reduced sexual affection | - Spousal discontent - Libido - Insomnia - Suicidal tendency - Disillusionment - Fear - Anxiety - Morbid thoughts - Insecurity - Social security - Disenchantment | P001” I cannot sleep in the night, and I don’t even know what to do. This world is a bitter place with incurable diseases. Hypertension is a problematic condition. When I think of the fact that I developed this disease at this young age, and no drug can cure it, I feel like killing myself. Hmmm it has not been easy for me at all.”  P005“I am always so weak, constant headache, and very fast heartbeat with difficulty to sleep at night, so sometimes I think killing myself will end all the suffering. It is better to die than to live with an illness which is not curable. I have to take drugs like that until I die.”  P014“As for me, I prefer to die and end it than to suffer like this. Anytime I am coming for review, they have to support me in the car because the stroke has affected my walking. I feel I am so much a burden to them (relatives). My son, it is not easy.”  P007“When I see my children coming, I pretend and smile like everything is ok; but inside me, I am a different person, always thinking of the future of my children, because if I get any complication like stroke now how do I take care of them, and this makes me so sad.”  P001“My brother, I have been thinking about why I should develop hypertension at this younger age. This means that I have to be on medication for the rest of my life, and this always makes me so sad.”  P008“A lot of things make me sad. Sometimes if I see a healthier younger woman of my age going about her normal activities, I frequently ask, why me? Why can’t I go up and down like them? Where did I go wrong to deserve this?”    P009“Hmmm, anytime I think of my death, I become afraid and anxious. I can’t imagine dying at this age and leaving my family behind. This puts so much fear in me because the children are quite young and who will take care of them when I am not there.”  P015“I am always anxious and afraid because of the media report I hear about hypertension. They always say once you are diagnosed with HPT; you cannot be treated in the hospital. And you will eventually get a stroke and die. Anytime I hear this kind of information from the radio stations I get frightened.”  P006“I have the fear that I may die. When I was diagnosed with hypertension, I cried because my aunty died from the complication of this same condition (HPT). Sometimes I am anxious about what will happen to me some days to come. I saw my aunty suffer a stroke as a result of HPT and eventually died”  P007,”I don’t have any urge for sex since I started taking the medication. I don’t have feelings for my wife, and I cannot remember the last time I made love with my wife. She is always complaining as if I have another woman elsewhere.”  P002“Things have changed, I used to disturb my wife for sex all the time, but now the urge is no more because of the constant palpitations I have, I don’t understand what is happening, my wife is always complaining!”  P005“(Laughs)…I have forgotten about that one. For sexual feelings, I have made up my mind because ever since I started taking the drugs, I don’t have the desire for sex. She has been complaining and even suggested that I go to see a doctor for treatment.” |
| **Socio economic factors** | - Loss of friends and social network - Difficulties with job demand - Financial burden | - Strained relations - Antisocial outcomes - Productivity - Health insurance - Financial aid - Cost burden - Debt | P006“These days I don’t regularly go to work because I have to always go for checkups on some of the days. This is affecting my productivity at the workplace. Because of that, my boss is always complaining and even threatening of sacking me.”  P008“I am always at home because when I walk small, I get tired. These days I usually don’t attend most of the activities that go on in this area, and because of that, I have lost most of my friends. I don’t get to see them, and they don’t come to visit me either.”  P001“Master, now my friends don’t come to me because I have not been visiting them these days because I don’t feel well. Not even one person since I felt sick. They have all abandoned me.”    P002“Now I don’t go to our usual joints to meet my friends for almost one year now because I easily get tired when I walk small so it is better, I stay at home, and they don’t visit me too at home. Some of them even think I am just lazy.”    P011 “At times, I don’t get the drugs in the hospital, and it is also expensive to buy the drugs outside the hospital. There are times that the drug is available in the hospital, but you have to pay an extra amount called top-up when you are an NHIS subscriber before you are given the medication. This brings about a substantial financial burden on me.”  P014 “Hmmm, as for my finances, it has affected me in so many ways. Anytime I go to collect drugs from the hospital, they always demand a top-up before I am served the medications though I am an NHIS subscriber. Everything in Ghana now is all about money.”  P012“I sometimes have to buy some of my prescribed medicines from the drug store (pharmacy) because it is not available in the hospital, and when I don’t have enough money, I have to borrow from someone and pay later.”  P009“As for the cost of treatment, it’s not easy at all. I have run into debt. Because when it was severe, we had to go to Sunyani regional hospital on referral to continue treatment. My money got finished at a point, and I had to borrow money to do all the laboratory investigations requested. They said the insurance does not cover some of the laboratory investigations, so I had to pay.”  P006” It is always difficult for me when it comes to my treatment because the doctors advised that I reduce my salt intake. So these days, my family has to prepare a different meal for me, and this also comes with extra costs.”  P002“In terms of money, since I started going to the hospital, I haven’t had any problem. Things are going on well with me because the family has been very supportive financially.”  P015 “All this while the private insurance company always pays the cost of treatment, and because of that, things are moving well with me. I don’t pay bills at the hospital when I go for treatment and reviews. The bills are always submitted to private insurance, and they pay on my behalf.” |
| **Coping strategies and support** | - Health system support - Social support - Religiosity | - adaptive behaviour - difficult schedule - social support; healthcare workers - service quality - social support: family - social support; religious - prayer - religious belief | P007 “The problem it has brought is that it has stopped me from doing my usual rounds. Typically, someone can call me that he or she needs an item to buy. I often quickly deliver the item. But these days, there are times that I have to reschedule the person to meet me at another time because I may be at the hospital because of my regular check-up, and this makes me lose my regular customers.”  P006“These days I don’t regularly go to work because I have to always go for checkups on some of the days. This is affecting my productivity at the workplace. Because of that, my boss is always complaining and even threatening of sacking me.”  P014 ”The health professionals have been very supportive in my treatment. They relate to me nicely, and some of them often give as health education on HPT, and I think they need to be applauded. Some even give me words of encouragement when they see that I am not looking cheerful, and this helps me to cope with the disease.”  P015“The staff always relate to me very nicely and very supportive. There are many changes among the staff members as compared to first. They are kind to us. As for me, I will be telling a lie if I say I have any problem with the staff of the hospital They take time to teach as on some of the things we need to do to stay healthy and the need for regular reviews.”  P009“My major problem is with the doctors of the hospital. They don’t spend time listening to our complaints. The moment you start to tell them about your problem, they will tell you that they are done with you, so go for drugs.”    P013“The problem I have is concerning the records office, you will sit down for long, and nobody will tell you anything, they shout at us as if we are children. The last time I nearly went back home because I sat for long and I was not getting my card number until one man came and told as the internet was off.”  P005 ”My wife’ is a perfect woman; she is always supporting and encouraging me all the time, and this makes me have a positive attitude. I think if you develop this condition and your wife doesn’t support you emotionally, you will die early.”  P004”“I am very grateful to God for my husband. He has helped me so much. He makes sure that he provides for me financially and even takes care of the home keeping. Our children are grown up, so we live alone. Truly, I wouldn’t have survived without him. We pray together, and he always encourages me to have faith in God.”  P001 ”My sister has been really supporting financially when it comes to payment of my hospital bills. She is always with me encouraging me, and that gives me a lot of strength to cope with this challenging disease because of the support she gives me”. At times she comes around to do cleaning and even cooks for me.”    P012“My family and friends have been very supportive ever since I was diagnosed with the disease (HPT). The extended family members visit me regularly, and some of them also support me financially.”  P006“My husband only buys food for me, but he does not have time for me. No affection, no attention, and care. He is always busy all the time and comes home very late”  P011“When I am not feeling well, as a husband, I expect him to spend meaningful time with me. At least, be close to me; hold me and talk with me and encourage me with words from the Quran. But he is always going to his second wife and leaves me to suffer alone.”  P015 ”Prayers is the key, and it has helped me. Sometimes it surprises me how God uses my prayers to make me move on. If not, constant prayers, I would have been dead by now. This sickness can easily kill you, but God has been good to me so far.”  P008“I have turned my house to be my chapel because I cannot go to church as often as I used to. And when you have a disease that has no cure, you have to turn to God for healing. My pastor and church leaders also visit me in my house regularly to pray with me; this helps some of as to cope with the disease.”  P013“It has made my faith stronger…I am much closer to God, and I believe this will bring an improvement in my disease situation, so I need to go on believing in God. I have the faith that I will get better one day, and this encourages me to move on.” |
