# Supplementary material for: Challenges experienced by patients with hypertension in Ghana: A qualitative inquiry
Source: PLoS One. 2021 May 6;16(5):e0250355. doi: 10.1371/journal.pone.0250355 (PMC8101754; doi:10.1371/journal.pone.0250355)
Supplement: S1 Table — (DOCX) [file pone.0250355.s003.docx]

**S1 Table**

**GENERAL PARTICIPANTS PROFILE**

| Codes | AGE | SEX | Educational  background | Religion | Marital  status | No. of children | Occupation | Language  spoken |
| --- | --- | --- | --- | --- | --- | --- | --- | --- |
| P001 | 36 | M | JSS | Christianity | married | 3 | Trader | Twi |
| P002 | 45 | M | diploma | Christianity | married | 4 | Teacher | Twi |
| P003 | 61 | M | None | Christian | married | 2 | Farmer | Twi |
| P004 | 50 | F | Diploma | Christian | married | 3 | Teacher | Twi |
| P005 | 38 | M | Diploma | Christian | married | 3 | Teacher | Twi |
| P006 | 55 | F | None | Christianity | married | 5 | Farmer | Twi |
| P007 | 53 | M | None | Christianity | married | 4 | Trader | Twi |
| P008 | 38 | F | Diploma | Muslim | married | 3 | Teacher | Twi |
| P009 | 61 | F | O’ level | Christianity | married | 3 | Fire service(rtd) | Twi |
| P010 | 41 | F | None | Muslim | married | 4 | cleaner | Twi |
| P011 | 56 | F | Primary | Muslim | married | 5 | Trader | Twi |
| P012 | 58 | F | Diploma | Muslim | Widow | 4 | Teacher | Twi |
| P013 | 60 | M | None | Christianity | married | 6 | Trader | Twi |
| P014 | 69 | M | Primary | Christianity | married | 5 | Retired | Twi |
| P015 | 58 | M | Primary | Christianity | married | 4 | cleaner | Twi |
